# Predictors of Suicide Attempts of Individuals with Autism and Their Siblings

**DOI:** 10.1155/2022/9157365

**Published:** 2022-09-02

**Authors:** Oren Shtayermman, Jason Fletcher

**Affiliations:** ^1^Chamberlain University, 500 Monroe St., Suite 28, Chicago, IL 60661, USA; ^2^NYU Rory Meyers College of Nursing, 433 First Ave., Room 735, New York, NY 10010, USA

## Abstract

The occurrence of mood- and anxiety-related conditions among family members of individuals diagnosed with autism spectrum disorder (ASD) increases the risk of suicide attempts and has also created assessment and treatment issues for clinicians and parents. Recorded rates of mental health disorders comorbid with suicide attempts among individuals on the spectrum range from 29% to 52%. The purpose of this study was to investigate the presence of predictors of suicide attempts among sibling dyads (pairs of siblings in which one sibling is on the autism spectrum). Growing evidence in the literature indicates a link between a diagnosis of autism and mental health. A web-based survey was used to collect data from individuals on the spectrum and their siblings. A sample of 144 individuals was finalized for the analyses. Logistic regression analysis was conducted to assess the predictors of suicide attempts in the sample. Depression, anxiety, sexual orientation, and marital status were all used as predictors in the logistic regression analyses. Both levels of anxiety and sexual orientation were found to be significant predictors for suicide attempts. Recommendations for future research, assessment, and treatment are presented.

## 1. Introduction

The rate at which autism spectrum disorder (ASD) is diagnosed has increased over the past two decades, and today, 1 in 54 persons is diagnosed with the condition [1]. According to the Diagnostic and Statistical Manual of Mental Disorders (5th edition), ASD is a complex neurodevelopmental disorder characterized by challenges in social communication and interaction [2]. Symptoms related to ASD vary from one person to another; some individuals may have difficulty maintaining social relationships, while others may be unsuccessful in responding appropriately in social settings. These deficits affect all domains of life, including school, work, and family relationships [3].

According to family systems theory, the family as a unit includes four subsystems: marital, parental, sibling, and extended family [4]. It would be beneficial for healthcare professionals to better understand the manner in which ASD influences all four subsystems within this framework, particularly siblings. Understanding these subsystems can assist practitioners in addressing the issues they may encounter. The documented challenges individuals on the spectrum face in relation to family functioning include communication, daily life, and relationships within the family [5]. The daily lives of individuals on the spectrum also impact their siblings. These siblings have described difficulties arising from a spectrum of behaviors, ranging from obsessive rituals and repetitive behaviors to impulsiveness. Outcome studies have shown mixed results regarding how siblings respond to their family members on the spectrum [3]. Although recent evidence has suggested that siblings of individuals with ASD may be at higher risk for psychological challenges similar to those experienced by the parents of such individuals, studies have reported contradictory findings about the link between having a sibling on the spectrum and the possible risk factors associated with this relationship [6, 7]. Previous research [8] has found that sibling dyads including individuals with autism where one sibling had a second disability had more asymmetric relationships (e.g., one sibling tended to dominate the other in an emotional sense). Furthermore, interactions between such pairs of siblings tended to become more asymmetric over time.

Most studies on siblings of persons with ASD have focused on children; few have evaluated adults [9]. Some of these studies have examined the manner in which siblings adjust to relationships [10], but none have focused on suicide as a risk factor. Furthermore, most past studies concerning the siblings of individuals with ASD have focused on infant siblings [11]. Aside from friendships and marriages/partnerships, sibling relationships are often the longest lasting of an individual's bonds with their relatives; although not all sibling relationships are close, some siblings may actively dislike one another, and in some cases cousins or other familial bonds may be closer than those between siblings in the same family [12]. The presence of an individual on the spectrum within a household can impact the adjustment and mental health of all members of that family, including siblings. Although the data we gathered suggest that the siblings of individuals with ASD are at higher risk for mental health issues [13], there is no indication that this risk is clinically significant [14]. Previous data suggest that siblings of individuals on the spectrum may internalize their challenges, including depression- and anxiety-related conditions [15]. Furthermore, because some siblings may eventually become caregivers and supporters of their siblings with ASD after their parents have passed, it is important to obtain a clearer understanding of such sibling dyad's lifelong journeys and mental health needs. The aims of this study were to estimate the rate of suicide attempts among individuals on the autism spectrum and their siblings and to identify the predictors of suicide attempts among individuals on the autism spectrum and their siblings.

### 1.1. Autism Spectrum Disorder and Suicide Attempts

Suicide is considered a global health crisis [16], and the current rate of suicide in the United States is 12.93 per 100,000 individuals, making it the 10th leading cause of death in the country. According to the Centers for Disease Control and Prevention (CDC), among people between the ages of 10 and 34 years, suicide is the fourth leading cause of death and occurs more than two and a half times more often than homicide. Gender and racial differences are well documented: women's suicide rates are reported to be highest for individuals between the ages of 45 and 64 years, and rates of suicide are highest among Native American and non-Hispanic males [17]. Individuals with disabilities have been neglected by and omitted from the focus of research. Specifically, individuals with neurodevelopmental disabilities are considered to be at a higher risk for suicide attempts [18]. In recent years, there has been a steady increase in the exploration and reporting of suicide among individuals diagnosed with ASD [19, 20]. Nevertheless, the exploration of suicidal behavior among individuals on the spectrum has encountered challenges, particularly the definition of suicide and methodological concerns at various research stages. Similar to ASD and the spectrum of symptoms associated with it, we can examine suicidal ideation and attempts on a spectrum from mild thoughts to death by suicide or completion, and there are currently more than 19 different definitions of suicide [21]. Concerns regarding intentionality, ideation, and behaviors all play a role in designing an exhaustive definition.

While the definition and diagnosis of ASD have received significant attention in the last few decades, they have also created challenges in understanding a condition that encompasses a variety of comorbidities [22]. The presence of mood- and anxiety-related conditions concurrent with suicide has created assessment and treatment issues for clinicians and parents. Rates of comorbid disorders among individuals on the spectrum range from 29% to 52% [23]. A recent meta-analysis of data on suicide among individuals on the spectrum indicated a rate of 7%–47% for suicide attempts, while that of suicidal ideation was 72% [24]. To date, only one study has examined the prevalence and risk factors for suicide attempts by siblings of individuals on the spectrum, and one other study from Sweden has examined deaths by suicide among individuals on the spectrum [25]. Because of the wide range of risk factors for suicide in the neurotypical population, the rate of suicide attempts and the risk of suicide among individuals with siblings on the spectrum need to be examined.

### 1.2. Risk Factors for Suicide Attempts

#### 1.2.1. Sociodemographic, Sexual Orientation, Employment, and Socioeconomic Status

Many studies have documented risk factors for suicide attempts in the general population; however, we still lack a consistent and accurate picture of suicide attempts among individuals on the spectrum and an even less accurate picture of their siblings. Walton [7] identified several sociodemographic factors related to the risk of externalizing behaviors among the siblings of a person diagnosed with ASD. These factors included family income, gender, and the age of the sibling diagnosed with autism. Due to the diversity of siblings of people with autism and the broad spectrum of symptom severity and methodological limitations [3], the data presented in this study should be considered alongside the framework of daily influences on development between siblings [26]. Research has found that some sociodemographic factors influence relationships between siblings, but there are no reports on the presence of psychopathology due to these variables, and lower levels of educational attainment and age have been most commonly associated with such relationships [9]. Identifying as lesbian, gay, bisexual, transgender, and queer (LGBTQ) has been shown to place an individual at higher risk for bullying and peer victimization as well as a range of mental health issues [27]. When assessing sexual orientation, a higher rate of homosexuality and bisexuality among individuals on the spectrum has been noted [28], making it an added risk factor for suicide. Mental health is influenced by a range of variables, including age, gender, race, and socioeconomic status. Individuals living in a home with a sibling on the autism spectrum may experience higher levels of stress [11]. Furthermore, these individuals may have to take over caring for a sibling on the spectrum in the event of their parents' passing or becoming unable to care for their children [29]. Reported data also indicate that the severity of autism and comorbid conditions increases the degree of concern regarding these individuals' mental health and adjustment [14], particularly with regard to depression diagnoses [15] and anxiety-related symptoms [30].

## 2. Methods

### 2.1. Study Design and Sample

This was a cross-sectional study examining suicide attempts, depression, and anxiety in individuals diagnosed with ASD and their siblings. Individuals registered with the Interactive Autism Network (IAN), an innovative online project that brings together many individuals who care for people with ASD and those impacted by it, were invited to participate in an online study based on a Qualtrics online survey. A total of 144 individuals diagnosed with ASD and their siblings completed the online survey. The eligibility criteria for the study were as follows: aged 18 or older, being diagnosed with ASD, and/or having a sibling registered with the IAN. Prospective participants were sent a letter describing the study and a link to complete the online survey. Before completing the survey, all individuals were asked to provide informed consent and were notified that the study was approved by a university institutional review board. We have created a survey and an informed consent at 8th grade level of education to ensure understanding of the language in the survey. The sample size of *n* = 144 yielded sufficient power (82%) to detect a small to medium effect size (adjusted odds ratio, AOR = 1.85) associated with a continuous predictor (depression and anxiety) in the primary analyses using logistic regression to predict history of a suicide attempt, based on a prevalence of suicide attempts of 17% and a 0.05 level of significance.

### 2.2. Measures

#### 2.2.1. Mental Health

The participants' mental health was assessed using the Beck Depression Inventory (BDI) and the Beck Anxiety Scale (BAI) [31, 32]. The BDI is a 21-item scale that can be self-administered by adults. The items on the scale, which relate to sadness, pessimism, loss of pleasure, and other such experiences, are scored from 0 to 3. The response categories correspond specifically to each domain. The scores range from 0 to 63, with higher scores indicating more severe depression symptoms. Scores above 20 indicate clinical depression, a score of 0–13 indicates minimal depression, 14–19 indicates mild depression, 20–28 indicates moderate depression, and 29–63 indicates severe depression. The internal consistency of the BDI has been rated as *α* = 0.91, and its one-week test–retest reliability is *r* = 0.93. In our sample, the BDI demonstrated strong interitem consistency (*α* = 0.93) and reliability (McDonald's *ω* = 0.93). The BAI is similarly structured, as it comprises 21 self-reported items and a list of anxiety-related symptoms. The participants were asked to indicate how much each symptom described had bothered them in the preceding week. Each symptom is rated on a four-point scale, ranging from 0 = not at all to 3 = severely. A score of 0–21 indicates low anxiety, 22–35 indicates moderate anxiety, and scores of 36 and above indicate potentially concerning levels of anxiety. The BAI showed high internal consistency (*α* = 0.92) and test–retest reliability over one week (*r* (81) = 0.75). It also demonstrated strong interitem consistency (*α* = 0.94) and reliability (McDonald's *ω* = 0.94) in the current study. The participants' history of suicide attempts was measured by asking them if they had ever attempted suicide. The response categories for this assessment were “yes” and “no.”

#### 2.2.2. Sociodemographic and Social Support

Sociodemographic data were obtained from the 144 individuals who completed the survey. The questionnaire was used to gather data related to age, ethnicity and race, and levels of education and income. The participants' levels of social support were measured using the Multidimensional Scale of Perceived Social Support (MSPSS) [33]. This 12-item measure comprises three subscales (support from family, friends, and significant others) designed to assess levels of social support. The *α* for the total scale was 0.88; for the subscales concerning significant others, family, and friends, the *α*s were 0.91, 0.87, and 0.85, respectively. Higher scores indicate higher levels of support [33]. In our sample, the MSPSS total score demonstrated strong interitem consistency (*α* = 0.94) and reliability (McDonald's *ω* = 0.93). The three subscales also had strong measurement characteristics: family (*α* = 0.93, McDonald's *ω* = 0.93), friends (*α* = 0.97, McDonald's *ω* = 0.97), and significant others (*α* = 0.95, McDonald's *ω* = 0.95).

### 2.3. Data Analysis

A descriptive summary was generated to describe the sample and the distribution of the study variables. Multivariate logistic regression was conducted to analyze the relationship between the independent variables (sociodemographic data, mental health, and social support) and the participants' histories of suicide attempts. Sensitivity analyses were conducted to examine the consistency of the findings between the persons with ASD and their siblings. This included subgroup analysis (persons with ASD) and testing interaction terms for significant predictors (interacted by sibling status). All analyses were conducted using SPSS version 22 (IBM, Armonk, NY, USA).

## 3. Results

The demographic characteristics of the sample are presented in Table 1. Of the 144 individuals who completed the study, 77.8% indicated that they were diagnosed with ASD, and 21.5% identified themselves as siblings of a person on the spectrum. The mean age of the sample population was 36.03 years (SD = 15.43). Most respondents were identified as Caucasian (86.1%) and heterosexual (72.2%), while less than half (38.9%) were male. Slightly less than half of the sample had completed an undergraduate degree (48.6%), and more than half were single (56.3%). The descriptive statistics related to mental health indicated that 17.4% had attempted suicide, 22.76% had experienced a high level of suicidal ideation, 13.9% had severe depression, and 14.53% had a potentially concerning level of anxiety. The individuals diagnosed with ASD reported higher rates of suicide attempts than the siblings of individuals with ASD (20.5% vs. 6.5%), but this difference was nonsignificant (*χ*^2^ (1, 143) = 3.33, *p* = 0.068).

Logistic regression was conducted to examine the relationship between the independent variables and the suicide attempts in the sample. The predictors of suicide attempts were selected based on previous findings and support from the current literature. These included clinical variables such as depression and anxiety and sociodemographic characteristics such as gender, marital status, sexual orientation, and social and employment characteristics. The predictors and covariates were entered in blocks. The first block consisted of anxiety and depression, followed by a block of the participants' demographic details (age, gender, race, sexual identity, and marital status) and finally a third block of environmental variables (education, social support, and personal income). The initial model, including only depression and anxiety, was significant, with anxiety significantly associated with a history of suicide attempts. The addition of covariates in the model strengthened the association, and the association between anxiety and suicide attempts was strengthened after controlling for participants' characteristics (Model 2), followed by the factors hypothesized to be protective (i.e., education, social support, and income).

The Hosmer–Lemeshow tests for each model were nonsignificant, indicating that the models had adequate goodness of fit. The *X*^2^ for the full model was significant (*X*^2^ (16, 114) = 53.20; *p* < 0.001). Its Nagelkerke's pseudo-*R*^2^ was 0.62, indicating that, as a whole, it was significant and that the combined predictors were associated with a history of suicide attempts. An examination of the regression coefficients (Table 2) indicated that the selected predictors were partially supported. Anxiety (AOR = 1.140, 95% CI 1.039–1.251, *p*=0.06) and sexual orientation (AOR = 0.020, 95% CI 0.002–0.170, *p* < 0.001) were significant predictors of a history of suicide attempts. These indicated that a 1-point increase on the BAI was associated with a 14% increase in the likelihood of having attempted suicide and that the heterosexual respondents were nearly 80% less likely to have done so compared to those who identified as nonheterosexual. Depression, age, gender, being a sibling of a person with ASD, marital status, education, employment status, social support, and income were not significantly associated with having attempted suicide.

As the sample was comprised of both individuals diagnosed with ASD and siblings of individuals with ASD, we conducted two sensitivity analyses to determine whether the associations identified were consistent across groups. First, we conducted an analysis excluding the sibling subgroup and compared the results to the analysis of the full sample. All model statistics and regression coefficients were consistent in magnitude, direction, and statistical significance. A similar analysis could not be done for only the siblings, as the group was too small to support the multivariate model. Second, focusing on the significant predictors of suicide attempts (anxiety and sexual orientation), we tested the interaction terms (sibling *∗* anxiety and sibling *∗* sexual orientation) in separate models. In these analyses, the interaction terms were nonsignificant, and their inclusion had no impact on the magnitude, direction, or significance of the predictors, indicating that the associations of anxiety and sexual orientation with suicide attempts do not differ between individuals with ASD and siblings of those with ASD.

## 4. Discussion

We examined the predictors of suicide attempts in a sample of sibling dyads, of whom one of each pair was an individual diagnosed with ASD. Specifically, we were interested in how sociodemographic variables such as age and gender, clinical variables such as depression and anxiety, and social variables such as employment status and social support can predict suicide attempts among sibling dyads, including individuals on the spectrum. Of the sample population evaluated in this study, approximately 77.8% indicated that they had been diagnosed with autism, and the average age of the participants was 36.03 years. Less than half of the sample identified as male, and the majority were Caucasian (72.2%). Similar to a previous study [20] in this field, this study indicated that approximately 14% of the sample reported a severe level of depression, 14.53% indicated that they had severe anxiety, and 22.76% indicated that they had attempted suicide at least once. These data are consistent with reports from the general population suggesting that Caucasian males are at higher risk: over 1.4 million individuals attempt suicide yearly, and there are 3.9 male deaths by suicide for each female death by suicide [34]. The presence and reports of mental health issues among sibling dyads that include individuals on the autism spectrum are important for screening and assessing family members (specifically siblings) who may present with mental health symptoms or diagnoses and are thus at high risk of suicide. The specific findings presented above indicate that, aside from certain sociodemographic characteristics that may place individuals at risk for suicide attempts, one of a pair of siblings being diagnosed with ASD can increase the risk of the other sibling developing a mental health condition and/or of attempting suicide [35]. In examining the risk factors for suicide attempts in sibling dyads that included individuals with autism, we identified two major predictors: anxiety and sexual orientation (specifically, identifying as nonheterosexual). We found that the individuals we surveyed were at a 12% higher risk for every added point on the BAI. This is an important outcome and is supported by previous studies on the general population, which have indicated that individuals with anxiety-related conditions are at a higher risk of suicide attempts [36]. The lack of predictability of life while living with a person on the spectrum and the potential of one day having to take over their care as parents age can cause or exacerbate anxiety and increase the risk of attempting suicide. With regard to those respondents who identified as nonheterosexual, our results are not consistent with previous reports related to the LGBTQ population in general. According to Peters et al. [37], individuals who are identified as nonheterosexual may present with more frequent or severe suicidal ideation, but this is not necessarily accompanied by higher rates of suicide attempts. This finding can be explained by the fact that services and support for autism may vary greatly from one person to another over a lifetime, which can add stressors and put individuals at risk of attempting suicide. Overall, the other variables included in the study were not significantly associated with a history of suicide attempts. This can be explained by several factors. First, our sample population was not evenly divided between individuals on the spectrum and their siblings, and our results may have been different if the two populations had been equally represented. Second, the individuals in our sample may have had protective variables that decreased their risk of attempting suicide. Protective factors for an individual could be social support, family support, or services received for a condition, to name a few. The multiple and vast presence of risk and protective factors for suicide can present a methodological challenge in accurately predicting a history of suicide attempts in sibling dyads that include individuals on the spectrum. Individuals' mental health and previous attempts should be assessed by anyone who provides professional care for families with these attributes to ensure that such risk factors are identified early and treated expeditiously.

### 4.1. Clinical Implications

A constant challenge for mental health clinicians and practitioners from various backgrounds is the need to determine whether an individual is at risk for suicide attempts early and to implement appropriate interventions. To date, no one particular model can predict with full accuracy the potential for a suicide attempt. Practitioners in a variety of settings can benefit from the data presented in this study, as they can provide points of reference and consideration for the further assessment of sibling dyads of individuals on the autism spectrum. The ability to identify symptoms related and potentially linked to suicide attempts early will benefit all practitioners. Supporting the comprehensive assessment and early identification of at-risk groups may make it easier to address the mental health needs of siblings of individuals on the spectrum. Moreover, the data reported in this study provide a framework to consider subunits within the family system (i.e., siblings), who should also be evaluated when individuals are seeking support services.

### 4.2. Research Implications

The increase in the number of studies that include family members of individuals on the spectrum provides a hopeful picture for many subgroups in this population, including parents, siblings, and community members. Engaging in further research on the impact autism has on family members can support families and healthcare professionals in providing person-focused services and treatment. A comprehensive examination of the mental health risks faced by siblings and other close family members of individuals with ASD can be used to design more effective support and treatment techniques. Perhaps more importantly, such an assessment could lead to a greater variety of more tailored services appropriate to this population, given their unique situation. Further investigations should consider multiple and varied assessment measures to exhaustively investigate the siblings of individuals on the spectrum and their challenges [38].

## 5. Limitations and Strengths

This particular survey was subject to recall bias, as the participants were asked about their thoughts over the preceding few weeks. Furthermore, the study did not include a control group of individuals with siblings who were not diagnosed with ASD. The lack of a control group may impact the manner in which we can respond to the needs of the siblings of individuals on the autism spectrum. Some methodological aspects of this study may impact its generalizability, including the use of an online survey and nonprobability sampling, as well as the utilization of the IAN as a tool to recruit individuals with the requisite eligibility criteria. The smaller subsample of siblings may also constitute a limitation, since the group of siblings and the group of individuals with a diagnosis of ASD was not equal. Despite these limitations, however, the study has multiple strengths. The participants were assessed for multiple risk factors for suicide attempts, and this study is one of the few to examine individuals on the autism spectrum and their siblings as a family unit. The use of the IAN also assisted us in reaching out to prospective participants across a larger geographical area.

## Figures and Tables

**Table 1:** Demographic characteristics of the sample (*N* = 144).

	Variable	*n* (%) or *M* (SD)
	Person Dx with ASD	112 (77.8%)
	Sibling of person Dx with ASD	31 (21.5%)
Age		36.03 (15.43)
Gender	Male	56 (38.9%)
	Female	86 (59.7%)
	Other	2 (1.4%)
Sexual orientation	Nonheterosexual	39 (27.1%)
	Heterosexual	104 (72.2%)
Race	Non-Caucasian	19 (13.2%)
	Caucasian	124 (86.1%)
Marital status	Single	81 (56.3%)
	Married	37 (25.7%)
	Other	26 (18.1%)
Education level	Undergraduate	70 (48.6%)
	Master	35 (24.3%)
	Other	38 (26.4%)
Employment	No	59 (41%)
	Yes	85 (59%)
Personal income	$20,000 or less	77 (53.5%)
	More than $20,000	63 (43.8%)
History of suicide attempts	No	119 (82.6%)
	Yes	25 (17.4%)
Mental health conditions	Depression	13.90 (12.38)
	Anxiety	14.53 (12.51)
	Social support	59.74 (17.10)

*Note.* Dx: diagnosed.

**Table 2:** Binary regression on variables that impact suicide attempts.

Variable	Model 1		Model 2		Model 3	
	*B* (SE)	OR (CI)	*B* (SE)	OR (CI)	*B* (SE)	OR (CI)
Depression	0.008 (0.024)	1.008 (0.962, 1.057)	−0.048 (0.037)	0.954 (0.887, 1.025	−0.078 (0.045)	0.93 (0.847, 1.010)
Anxiety	0.078 (0.026)^†^	1.081 (1.028, 1.137)	0.102 (0.038)^†^	1.108 (1.028, 1.193)	0.131 (0.047)^†^	1.14 (1.039, 1.251)
Age			−0.006 (0.027)	0.994 (0.942, 1.049)	−0.028 (0.036)	0.973 (0.907, 1.043)
Sibling of person Dx with ASD (ref: person Dx with ASD)			−0.970 (1.171)	0.379 (0.038, 3.763)	−1.123 (1.213)	0.325 (0.030, 3.505)
Gender (ref: male and other)			−1.049 (0.862)	0.35 (0.065, 1.897)	−1.391 (1.021)	0.249 (0.034, 1.839)
Sexual orientation (ref: non-heterosexual)			−3.672 (0.943)^‡^	0.025 (0.004, 0.161)	−3.925 (1.010)^‡^	0.02 (0.002, 0.170)
Race (ref: non-caucasian)			0.317 (0.945)	1.374 (0.215, 8.756)	0.708 (1.066)	2.029 (0.251, 16.408)
Marital status (ref: other arrangement)						
Single			−1.966 (0.992)^*∗*^	0.14 (0.020, 0.979)	−1.900 (1.205)	0.15 (0.014, 1.586)
Married			1.040 (0.961)	2.829 (0.431, 18.596)	1.802 (1.154)	6.061 (0.632, 58.172)
Education (ref: other)						
Undergraduate					−0.654 (1.129)	0.52 (0.057, 4.757)
Master					−0.742 (1.120)	0.476 (0.053, 4.280)
Employed (ref: not employed)					−0.073 (1.035)	0.929 (0.122, 7.071)
Personal income					0.622 (1.106)	1.863 (0.213, 16.298)
Social support*p* < 0.001-significant other					−0.496 (0.328)	0.609 (0.320, 1.159)
Social support-family					−0.060 (0.311)	0.941 (0.512, 1.731)
Social support-friend					0.016 (0.316)	1.016 (0.547, 1.885)

*Note*. ^*∗*^*p* < 0.05; ^†^*p* < 0.01; and ^‡^*p* < 0.001. Reference group: gender: male; marital status: other arrangements (i.e., divorced, widowed, separated, living with partner, or single); race: non-Caucasian; sexual orientation: nonheterosexual. B: coefficient; CI: 95% confidence interval; Dx: diagnosed; OR: odds ratio; and ref: reference group.

## Data Availability

The data that support the findings of this study can be obtained from the corresponding author upon request. The data are not publicly available due to privacy or ethical restrictions.
